# The complete chloroplast genome sequence of *Bambusa contracta* L.C.Chia & H.L.Fung (Bambusodae)

**DOI:** 10.1080/23802359.2021.1944361

**Published:** 2021-06-28

**Authors:** Yinghui Zhang, Yangyang Zhang, Muhammad Waqqas Khan Tarin, Jalal Hayat Khan, Tianyou He, Yushan Zheng

**Affiliations:** a Fujian Vocational College of Agriculture, Fuzhou, Fujian, PR China; b College of Forestry, Fujian Agriculture and Forestry University, Fuzhou, Fujian, PR China; c College of Landscape Architecture, Fujian Agriculture and Forestry University, Fuzhou, Fujian, PR China; d Rangeland Research Institute, National Agricultural Research Centre, Islamabad, Pakistan

**Keywords:** *Bambusa contracta*, Bambusodae

## Abstract

*Bambusa contracta* L. C. Chia & H. L. Fung is widely distributed in the foothills of Guangxi province, China, and used as a raw material for the production of various bamboo weaving products. In the present work, the complete chloroplast genome sequence of *B. contracta* was characterized by Illumina pair-end sequencing. The chloroplast genome of *B. contracta* was 139,470 bp in total length, containing a large single-copy (LSC) region of 83,187 bp, a small single-copy (SSC) region of 12,897 bp, and two inverted repeats (IR) regions of 21,693 bp. The genome consisted of a total of 127 genes, with 83 protein-coding genes, 36 tRNA genes, and eight rRNA genes. Based on 14 chloroplast genomes, the phylogenetic analysis revealed that *B. contracta* is closely related to *B. emeiensis* in Bambusodae.

*Bambusa contracta* L.C.Chia & H.L.Fung (*B. contracta*) is mainly distributed in the foothills of Guangxi province, China, and is used as raw material for the production of various bamboo weaving products. The poles of the *B. contracta* are 5–6 m taller, 2–3 cm in diameter, 40–57 cm in internodes length, and the tip of the tail is curved, while the lower portion is straight (http://powo.science.kew.org/taxon/urn:lsid:ipni.org:names:897025-1). The chloroplasts (cp) genome has a maternal ancestry and symmetrical arrangement which is used to determine the developmental and phylogenetic relationship of plants (Wang et al. 2018). In this study, we first assembled the complete cp genome of *B. contracta* based on Illumina pair-end sequencing data. We collected the 50 g leaf samples of *B. contracta* from Fujian Province, China (University of Fujian Agriculture and Forestry, Bamboo Garden, Fuzhou: 119°14′16″E, 26°5′7″N) and dried them immediately with silica gel for DNA extraction. In addition, the certificate specimens were deposited in the Bamboo Research Institute, Fujian Agriculture and Forestry University (contact person name: Tianyou He, Email: hetianyou@fafu.edu.cn) under the voucher number 101303. Fresh leaves of the individuals were collected and flash-frozen in liquid nitrogen and then stored in a refrigerator (–80 °C) until DNA extraction. After DNA extraction, its quantitative measurements were authenticated by employing Agarose gel electrophoresis and Nanodrop concentration, 500 bp randomly interrupted by Covaris ultrasonic breaker for library construction. Approximately 2.0 GB of raw data were generated with 150 bp paired-end read lengths. The Illumina High-throughput sequencing platform (HiSeq 2500) data were filtered by the script in the NOVOPlasty (Dierckxsens et al. 2017). The complete plastid genome of *Bambusa arnhemica* (GeneBank accession: KJ870989) as a comparison and plastid genome of *B. contracta* were assembled by GetOrganelle pipeline (https://github.com/Kinggerm/GetOrganelle), it can get the plastid-like reads, and the reads were viewed and edited by Bandage (Wick et al. 2015). The cp genome annotation was assembled based on the comparison by Geneious v 11.1.5 (Biomatters Ltd, Auckland, New Zealand) following Kearse et al. (2012).

The complete chloroplast genome sequence of *B. contracta* (GenBank number: MW190088) was characterized by Illumina pair-end sequencing. Raw reads were deposited in the GenBank Sequence Read Archive (SRA; PRJNA692670). The cp genome of *B. contracta* was 139,470 bp in length, which was composed of four distinct regions such as a large single-copy (LSC) region of 83,187 bp, a small single-copy (SSC) region of 12,897 bp, and a pair of inverted repeats (IR) regions of 21,693 bp. The complete cp genome consisted of 127 genes, including 83 protein-coding genes, 36 tRNA genes, and eight rRNA genes. The complete cp genome GC content was 38.90%. In order to characterize the phylogenetic position of *B. contracta* with other members of Bambusodae, we conducted a phylogenetic analysis focusing on 13 complete cp genomes of Bambusodae and one taxa (*Ampelocalamus calcareus*) as outgroups which were downloaded from NCBI GenBank. The sequences were synchronized with MAFFT v7.307 (Katoh and Standley 2013), and the phylogenetic tree was constructed by RAxML (Stamatakis 2014). Both the *B. contracta* and *B. emeiensis*, belonging to the Bambusodae family, had a close relationship. The analysis of the cp genome of *B. contracta* provides excellent genetic information for further studies of this precious species and the taxonomy, phylogenetics, and evolution of Bambusodae (Figure 1).

**Figure 1.**
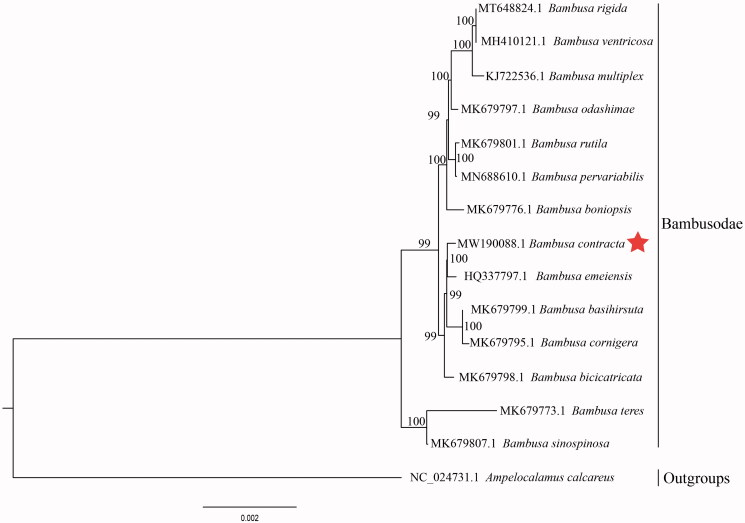
Phylogenetic analysis of 14 complete cp genomes of Bambusodae, and one taxa (*Ampelocalamus calcareus*) as outgroups based on plastid genome sequences by RAxML, numbers close to each node are bootstrap support values.

## Data Availability

The data that support the findings of this study are openly available in GeneBank (https://www.ncbi.nlm.nih.gov/). The complete chloroplast genome generated for this study has been deposited in GeneBank with accession number MW190088 (BankIt 2394036 101303). All high-throughput sequencing data files are available from the GenBank Sequence Read Archive (SRA) accession number: PRJNA692670.
